# Cost and Impact of Dried Blood Spot Versus Plasma Separation Card for Scale-up of Viral Load Testing in Resource-limited Settings

**DOI:** 10.1093/cid/ciz338

**Published:** 2019-04-26

**Authors:** Brooke E Nichols, Sarah J Girdwood, Aaron Shibemba, Sharper Sikota, Christopher J Gill, Lawrence Mwananyanda, Lara Noble, Lynsey Stewart-Isherwood, Lesley Scott, Sergio Carmona, Sydney Rosen, Wendy Stevens

**Affiliations:** 1 Department of Global Health, School of Public Health, Boston University, Massachusetts; 2 Health Economics and Epidemiology Research Office, Department of Internal Medicine, School of Clinical Medicine, Faculty of Health Sciences, University of the Witwatersrand, Johannesburg, South Africa; 3 Ministry of Health, Lusaka; 4 Right to Care Zambia, Lusaka; 5 Liverpool John Moores University, United Kingdom; 6 Department of Molecular Medicine and Haematology, Faculty of Health Sciences, University of the Witwatersrand; 7 National Health Laboratory Service, Johannesburg, South Africa

**Keywords:** viral load, dried blood spot, plasma separation card, cost modeling, geospatial modeling

## Abstract

**Background:**

Routine plasma viral load (VL) testing is recommended for monitoring human immunodeficiency virus–infected patients on antiretroviral therapy. In Zambia, VL scale-up is limited due to logistical obstacles around plasma specimen collection, storage, and transport to centralized laboratories. Dried blood spots (DBSs) could circumvent many logistical challenges at the cost of increased misclassification. Recently, plasma separation cards (PSCs) have become available and, though more expensive, have lower total misclassification than DBSs.

**Methods:**

Using a geospatial model created for optimizing VL utilization in Zambia, we estimated the short-term cost of uptake/correct VL result using either DBSs or PSCs to increase VL access on equipment available in-country. Five scenarios were modeled: (1) plasma only (status quo); (2) plasma at high-volume sites, DBS at low-volume sites; (3) plasma at high-volume sites, PSC at low-volume sites; (4) PSC only; (5) DBS only.

**Results:**

Scenario 1 resulted in 795 342 correct results due to limited patient access. When allowing for full and partial adoption of dried specimens, access increases by 19%, with scenario 3 producing the greatest number of correct results expected (929 857). The average cost per correct VL result was lowest in the plasma + DBS scenario at $30.90 compared to $31.62 in our plasma + PSC scenario. The cost per correct result of using dried specimens only was dominated in the incremental analysis, due primarily to fewer correct results.

**Conclusions:**

Adopting the partial use of dried specimens will help achieve improved VL access for patients at the lowest cost per correct result.

## Teaser

Use of dried specimens can increase the number of patients accessing viral load (VL). Adopting partial use of dried specimens will help achieve improved VL access for patients at a lower cost per correct result than complete adoption of dried specimens.

Routine viral load (VL) testing is the World Health Organization–recommended method for monitoring human immunodeficiency virus (HIV)–infected patients on antiretroviral therapy (ART), and many countries are rapidly scaling up VL testing capacity [1]. Zambia, a high-HIV-prevalence country in southern Africa, conducts VL tests on plasma specimens, which is considered the gold standard for VL testing.

We previously developed a geospatial mathematical model to assist the scale-up of VL testing in Zambia [2]. The first application of this model was to create an optimized VL sample transportation network for the transfer of plasma specimens from points of service delivery (clinics) to centralized laboratories. Programmatic implementation of the optimized sample transportation network model, however, has encountered logistical challenges: Some facilities cannot be reached for sample pickup as often as expected (or at all), and some samples cannot be processed due to breaks in the cold chain or delayed centrifugation, or are damaged during transport. As a result, utilization of VL testing at laboratories has been much lower than the model predicted. These issues are also sensitive to economies of scale around the logistical challenge of drawing, storing, and transporting blood transport samples to a VL laboratory within 24 hours of blood draw. As a consequence, patients at low-volume health facilities are less likely to have a VL processed successfully than patients at high-volume facilities due to requirements of drawing, storing, and transporting plasma.

Many of these challenges relate to the use and transportation of plasma samples, which require centrifugation and cold storage. Dried specimen use is expected to address some of these challenges and improve access to and uptake of VL testing: No refrigeration is required, blood can be drawn irrespective of sample transport schedules, and blood can be obtained more easily by either fingerstick or heel-prick specimens. Dried specimen use also has the benefit of creating a more patient-centered approach to clinical monitoring, as dried specimens can be collected any day of the week, whenever the patient comes to the clinic.

Although dried blood spot (DBS) testing is a widely used sample collection method in neighboring countries [3] and is used for early infant diagnosis in Zambia, it has not yet been approved for routine VL testing in Zambia. The potential disadvantages cited for adoption of DBS include decreased test sensitivity and specificity at the defined threshold of 1000 copies/mL and, depending on the platform, the possibility of increased laboratory staff time required to process the samples, placing extra demands on already constrained human resource capacity [4, 5]. Yet potential advantages are many, and include improved access to VL testing and decreased costs related to sample transportation, particularly in remote or low-patient-volume settings where the feasibility of daily collection of whole blood or plasma samples is logistically challenging. Recently, dried plasma spot (DPS) technology using plasma separation cards (PSCs) has become available and is acceptable for VL testing [6]. Though more expensive to procure than DBS per test kit, PSC technology is showing improved sensitivity and specificity compared to DBS [7].

In this analysis, we extend our existing geospatial model of VL scale-up in Zambia to estimate the impact, in terms of numbers of accurate tests completed and related costs, of complete or partial adoption of DBS or PSC for VL sample collection in Zambia.

## METHODS

### Model Design

**Figure 1.**
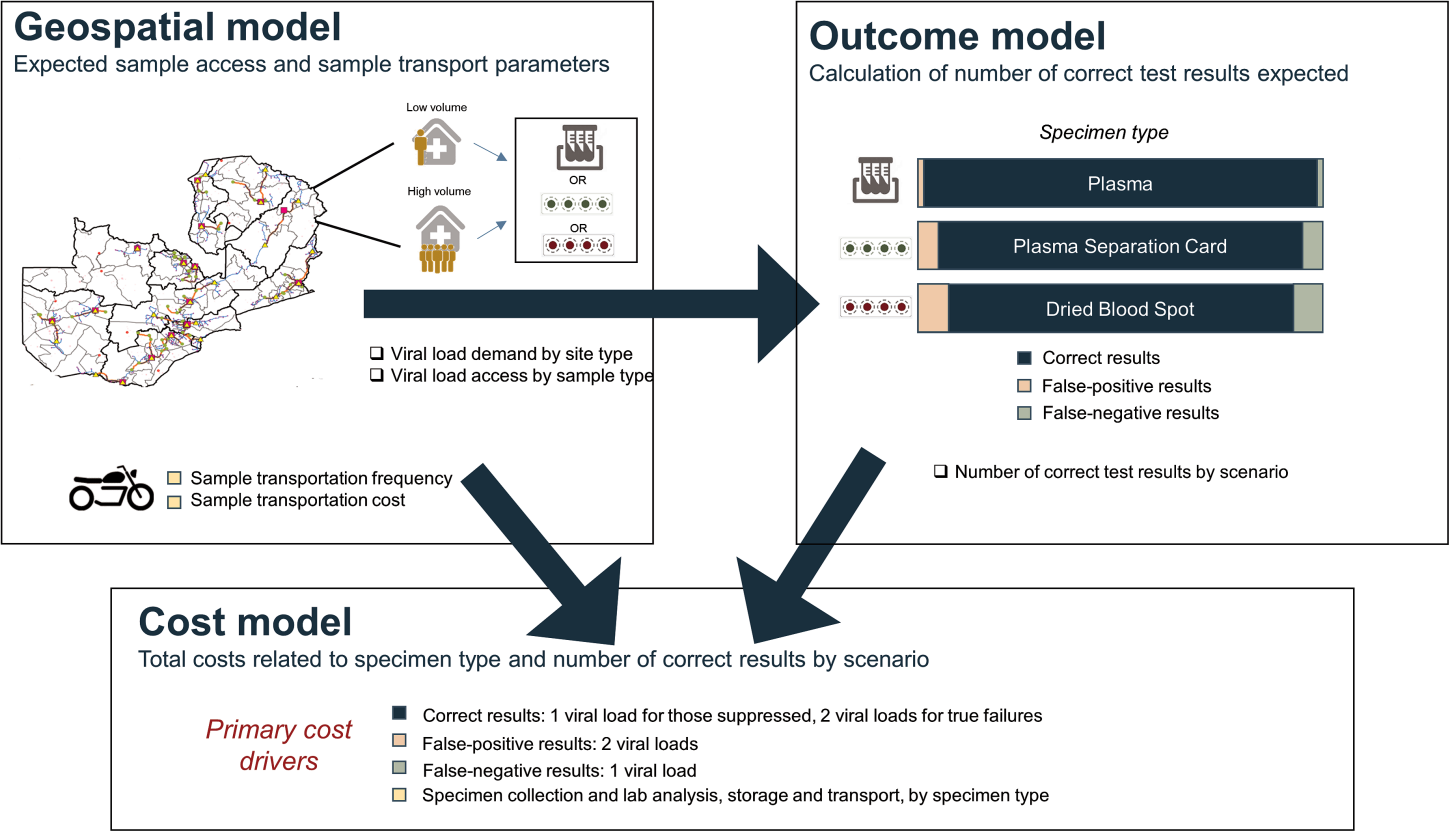
Schematic of the integration of geospatial model with a cost-outcomes model for determining the cost and impact of plasma, plasma separation card, or dried blood spot samples.

The objective of this analysis was to estimate the cost per correct VL result of plasma and dried specimens (both DBS and PSC) for VL sample collection. To do this, we included output from our previously described geospatial model, created to optimize VL scale-up in Zambia, in an analytic cost and outcomes model (Figure 1) [2]. The previously optimized sample transportation network connected a total of 152 high-volume facilities to a VL laboratory on a daily basis, with high volume defined as an anticipated ≥10 VLs per facility per day and represent 70% of the total anticipated 2020 testing volume. An additional 648 low-volume facilities (anticipated <10 VLs per day), representing 21% of the anticipated 2020 testing volume, were reached weekly.

Five scenarios were modeled:

1. Base case: full implementation and expected utilization of plasma-only VL monitoring (“plasma only”);
2. Partial adoption: plasma at high-volume sites and DBS at low-volume sites (“plasma + DBS”);
3. Partial adoption: plasma at high-volume sites and PSC at low-volume sites (“plasma + PSC”);
4. Full system switch to DBS (“DBS only”);
5. Full system switch to PSC (“PSC only”).

The total number of correct results expected was calculated based on the number of tests expected to be conducted in each scenario and the test accuracy reported in Table 1.

**Table 1.** Sensitivity and Specificity, by Specimen Type

Specimen Type	Equipment	Expected	Low	High	Source
Sensitivity					
Plasma	Roche (CAP/CTM)	0.983	0.967	0.993	[8]
DPS + PSC	Roche (CAP/CTM)^a^	0.91	0.84	0.96	[7, 9]
DPS + PSC	Cobas 8800^a^	0.970	0.924	0.992	[7]
DBS	Roche (CAP/CTM)^a^	0.948	0.846	0.984	[4, unpublished data]
Specificity					
Plasma	Roche (CAP/CTM)	0.994	0.983	0.999	[8]
DPS + PSC	Roche (CAP/CTM)^a^	0.99	0.98	1	[7, 9]
DPS + PSC	Cobas 8800^a^	0.972	0.949	0.986	[7]
DBS	Roche (CAP/CTM)^a^	0.939	0.720	0.989	[4, unpublished data]

Abbreviations: CAP/CTM, Roche Cobas Ampliprep/Cobas TaqMan; DBS, dried blood spot; DPS, dried plasma spot; PSC, plasma separation card.

^a^Threshold of 1000 copies/mL used.

### Expected Patient Volumes

Expected patient volumes were calculated by assuming different sample loss/uptake multipliers dependent on the specimen and facility type. These multipliers were calculated using programmatic data from 3 provinces in Zambia (collected by the in-country implementing partner, EQUIP Health) where the optimized sample transportation network is currently being rolled out. These data showed that low-volume sites were only requesting half the number of VL test volumes compared to high-volume sites per number of patients currently on ART (10% vs 20% annual patient coverage at a facility in 2 of 10 Zambian provinces as of March 2018). To model full-scale up, high-volume sites were assumed to have a maximum of 80% of patients accessing plasma VL annually, with the remaining 20% assumed to encounter problems with providing/drawing blood samples, cold chain storage, demand creation, and/or sample transportation that resulted in sample degradation. As a conservative estimate, we assumed the 80% of annual patient access would persist for high-volume sites with the use of DBS/PSC.

For low-volume facilities in the plasma-only collection scenario, we assumed that 40% of patients would access VL testing annually. This was increased to 80%, equivalent to high-volume facilities, with the adoption of dried specimens as providers/patients could access VL sample collection services every day, and sample degradation may also decrease. This increase in patients’ accessing VL at low-volume facilities represents an additional 16% of patients accessing VL tests annually. There are also facilities that cannot be reached at all for plasma sample transport due to distances/transport travel time that exceed allowable time for whole blood at ambient temperatures. In the dried specimen scenarios, therefore, we also included additional facilities that cannot be reached in the plasma-only scenario, which results in an additional 3% of patients accessing VL tests annually. The combination of improved access at existing facilities and expanded access to new facilities resulted in a projected national 19% increase in VL access in the dried specimen scenarios.

### Existing and Planned VL Testing Equipment and VL Testing Performance

VL testing is currently centralized at 19 laboratories across Zambia. Equipment currently utilized includes the Roche Cobas Ampliprep/Cobas TaqMan 48 and the Roche Cobas Ampliprep/Cobas TaqMan 96 (CAP/CTM), and the Cobas 4800 system (Cobas) (Roche Molecular Diagnostics, Branchburg, New Jersey). For this analysis, we explored the use of the recently launched Roche PSC on both the CAP/CTM and Cobas as an alternative to DBS on the CAP/CTM. The sensitivity and specificity of PSC was applied to the respective volumes of CAP/CTM and Cobas expected by the sample transport system: 42% on CAP/CTM in the partial adoption scenario, and 61% on CAP/CTM in the full adoption scenario. We assume that DBS sample preparation on CAP/CTM will be done using the free virus elution protocol, given its higher sensitivity and specificity compared to the specimen preextraction reagent sample preparation methodology [5, 10, 11].

### Cost Inputs

Costs included were (1) the sample collection costs by specimen type (Supplementary Tables 1–5); (2) sample transport network costs by scenario; (3) the cost of a visit for VL results; and (4) the all-inclusive cost per VL test conducted at a centralized laboratory (Table 2 and Supplementary Text 1). The cost per patient with a VL result (total costs incurred for all VLs divided by the number of patients accessing VL) and the cost per patient with correct result by scenario (total costs incurred for all VLs provided divided by the number of patients provided with a correct result) is reported. To compare each scenario to the current plasma-only status quo, we calculated the (1) average cost per additional patient with correct VL result for each respective dried specimen scenario compared to the plasma-only scenario, and (2) incremental cost per additional patient with correct VL results, where each scenario is compared to the next least costly scenario.

**Table 2.** Cost Inputs and Cost per Viral Load Test, by Specimen Type^a^

Cost Input	Value (US$)	Source	
Visit cost to facility	3.65	[13]	
Monitoring costs			
Viral load testing using the Roche platform (US$)	Plasma	DBS	PSC
Sample collection consumables	0.31	0.92	5.00^b^
Sample collection staff, equipment, and overhead	0.87	0.47	0.47
Sample analysis at laboratory	17.22	17.54	17.54
Total cost per viral load test	18.40	18.93	23.01

Abbreviations: DBS, dried blood spot; PSC, plasma separation card.

^a^Excludes any cost of reporting results to facilities after sample analysis completed.

^b^Based on personal email communication with Roche (24 January 2019).

### Sensitivity Analysis

To assess the robustness of our model and conclusions, we conducted a multiple univariate sensitivity analysis of the key inputs. We calculated the annual cost per patient with correct VL results for the partial adoption scenarios for changes in the cost of the PSC collection kit, cost of VL testing on a Roche platform, patient access rate, sensitivity and specificity of DBS and PSC, the true underlying proportion of patients with virological failure, and a reduction in repeat VLs. We then conducted a bivariate analysis by simultaneously varying the values of 2 of the key input variables.

## RESULTS

The modeled plasma-only system reaches 814 066 patients annually at a cost per person accessing VL of $29.23 and cost per person with a correct VL result of $29.92 (Table 3). When allowing for the full and partial adoption of dried specimens, access increases by 19% to 965 587 patients. Of those 965 587 patients, 74% (708 525) are located in high-volume facilities that would make use of plasma samples and daily sample transport, and 26% (257 062) are located in low-volume facilities that would make use of either DBS or PSC and biweekly transport in the partial adoption scenarios.

**Table 3.** Total Access, Number of Correct Viral Load Results, and Cost of Using Combinations of Plasma and Dried Specimens for Viral Load Sample Collection in Zambia

	Potential Access With Only Plasma Specimen Use		Potential Access With Dried Specimen Use							
	Plasma Only		Plasma High Volume + DBS Low Volume		Plasma High Volume + PSC Low Volume		DBS Only For Whole VL System		PSC Only for Whole VL System	
	No.	Cost	No.	Cost	No.	Cost	No.	Cost	No.	Cost
No. of patients accessing VL tests/y (% total)	814 066 (65%)	…	965 587 (77%)	…	965 587 (77%)	…	965 587 (77%)	…	965 587 (77%)	
No. of ART facilities reached (% total)	800 (54%)	…	1041 (71%)	…	1041 (71%)	…	1041 (71%)	…	1041 (71%)	…
No. of patients with correct VL results	795 342	…	920 243	…	929 857	…	856 476	…	884 844	…
VLs on dried specimens	…	$0	306 860	$5 808 856	296 308	$6 818 046	1 152 640	$21 819 481	1 108 200	$25 499 686
VLs on plasma specimens	930 982	$17 130 067	810 283	$14 909 215	810 283	$14 909 215	…	$0	…	$0
No. of facility visits required	931 011	$3 398 191	1 117 143	$4 081 157	1 106 591	$4 039 542	1 152 640	$4 220 251	1 108 200	$4 045 952
Transport cost	…	$3 264 509	…	$3 637 168	…	$3 637 168	…	$2 390 317	…	$2 390 317
Annual system cost	…	$23 792 767	…	$28 436 396	…	$29 403 970	…	$28 430 050	…	$31 935 954
Cost per patient with a VL result	…	$29.23	…	$29.45	…	$30.45	…	$29.44	…	$33.07
Cost per patient with correct VL results	…	$29.92	…	$30.90	…	$31.62	…	$33.19	…	$36.09
Average annual cost per additional patient with correct VL results compared to plasma only	…	…	…	$37.18	…	$41.71	…	$75.85	…	$90.98
Incremental annual cost per additional patient with correct VL results	…	…	…	$37.18	…	$100.64	Weakly dominated		Dominated	

Abbreviations: ART, antiretroviral therapy; DBS, dried blood spot; PSC, plasma separation card; VL, viral load.

The plasma + PSC scenario correctly classifies the greatest number of VL results: 929 857 compared to 920 243 in the next best scenario (plasma + DBS). The average cost per correct VL result, however, was lowest in the plasma + DBS scenario at $30.90 compared to $31.62 in our plasma + PSC scenario. The average cost per additional patient with a correct VL result as compared to the plasma-only scenario is lowest for plasma + DBS scenario at $37.18 followed by plasma + PSC at $41.71. The incremental annual cost per additional patient with a correct result in the plasma + PSC scenario relative to the plasma + DBS scenario is $100.64.

While access remains the same, the cost per patient with a correct VL result is the highest in the dried specimen–only scenarios, $33.19 and $36.09 for the DBS-only and PSC-only scenarios, respectively, due to fewer number of correct results (average cost per additional patient with a correct VL result at $75.85 and $90.98, respectively). In the incremental analysis, these scenarios were dominated by the partial dried specimen scenarios.

The cost of the PSC sample collection kit was a key input parameter in our sensitivity analysis (Supplementary Text 2). With a reduction in the PSC collection kit price from $5 to $2.72, plasma + PSC would cost the same as plasma + DBS per correct result ($30.90), and a reduction to $1.72 for the PSC collection kit would result in cost neutrality for the plasma + PSC compared with plasma + DBS scenario in terms of cost per patient with a VL result. Our results were sensitive to the performance of the specimen type in the plasma + PSC scenario and the plasma + DBS scenario, the underlying rate of virological failure, and the Roche dried specimen testing cost (Supplementary Figure 1). The plasma + DBS scenario is very sensitive to a decrease in the specificity of DBS: A deterioration in the specificity of DBS from the expected value of 94% to 89% would result in the plasma + PSC being cost neutral relative to the plasma + DBS scenario. Similarly, a decrease in the sensitivity of DBS from the expected value of 95% to 85% renders the plasma + PSC cost neutral to the plasma + DBS scenario.

## DISCUSSION

For many low- and middle-income countries, dried specimen use at low-volume and remote facilities provides a simple mechanism to improve VL access and uptake and thus patient outcomes. We found that a partial adoption of dried specimens resulted in the greatest number of patients with correct VL test results in Zambia. The price of the PSC kit would have to be reduced to be considered cost-neutral to partial DBS adoption in the short-term. However, as changes in sensitivity/specificity of DBS and PSC change the conclusions of the partial adoption scenarios, the sensitivity and specificity of DBS vs PSC in practice should be assessed in the field, as this is the key driver of difference in cost between the 2 specimen collection technologies.

There will clearly be additional short-term costs incurred in improving VL access. Improving access to VL testing justifies this short-term investment, however, by reducing the potential for misclassification of patients as failing or suppressed when they are monitored clinically. VL results are also typically required for a patient to become eligible for differentiated, and often less burdensome, models of ART service delivery. These differentiated models of care typically require fewer visits to the facility and, as such, improved VL access may also reduce the cost of treating a subset of patients. Finally, partial adoption of dried specimens provides 16%–17% more patients with correct VL results, and 30% more facilities with VL access.

A number of studies have evaluated the performance of DBS, DPS, and plasma for VL testing across a number of platforms [13–16]. A modeling study of the cost-effectiveness of patient monitoring strategies has shown VL using DBS to be the most cost-effective monitoring strategy compared to clinical monitoring and CD4-based monitoring [17]. These earlier analyses did not, however, consider DPS using PSC as a competing alternative. To our knowledge, this is the first study to model the costs and impact of using different specimen types and technologies, including PSC, on access and utilization in a country-wide VL monitoring program in a resource-limited setting, with primary data on laboratory and facility location data matched to programmatic ART data. The analysis relies on an innovative geospatial optimization model of VL scale-up in Zambia, which has been used to identify facilities and patient populations for whom dried specimen adoption would be beneficial [2].

There are several limitations to this study. First, we may have underestimated the difference in misclassification between DBS and PSC. We used the results of a meta-analysis of DBS and compared that to the one laboratory-based study of PSC available. Estimates of assay performance from different populations with different underlying distributions of VL failure are not directly comparable. Further studies are needed to evaluate the performance of both DBS and PSC in a head-to-head analysis on the same equipment in the field on the same samples. In the absence of such data, we used estimates from the analysis of PSC in South Africa where the same samples were tested using both DBS and PSC with DBS being performed on the Abbott rt2000 platform and PSC being performed on the Cobas 6800/8800 and CAP/CTM [7]. While this is not a perfect head-to-head analysis (DBS is not conducted on the Cobas 6800/8800 or CAP/CTM), by using the same population with the same underlying distribution of VL failure, it is a better comparison. Results from this analysis significantly strengthen the case for the partial adoption of PSC, with the plasma + PSC scenario outperforming the plasma + DBS scenario (Supplementary Text 3 and Supplementary Tables 6 and 7). This was not included in our primary analysis as we wanted to use only the equipment available in Zambia. Second, this analysis has utilized the current equipment installed in Zambia, the Roche CAP/CTM platform and the Cobas, to evaluate the partial and full adoption of DBS vs PSC. Extrapolation of these findings to other countries would need to take into account the platforms in use in those countries and the sensitivity and specificity of different specimen types on those platforms. For example, DBS performance on an Abbott platform has historically outperformed DBS performance on a Roche platform [13, 14]. Recently, however, DBS sensitivity/specificity using the free virus elution sample preparation protocol on the Roche CAP/CTM (94.8% sensitivity, 93.9% specificity) closely approaches the DBS performance on the Abbott RealTime platform (88.3% sensitivity, 99.1% specificity) [4, unpublished data]. Since the Roche CAP/CTM and Cobas are already available in Zambia, the findings of this study will provide direct guidance to the Zambian Ministry of Health in expanding VL access. Zambia may introduce the Cobas 6800/8800 in Zambia more widely, which would result in a higher PSC sensitivity (outweighing the costs as a result of the reduction in specificity) and therefore strengthen our conclusions. Third, this analysis explored the use of dried vs plasma specimens to increase VL access and uptake using Zambia’s existing laboratories and sample transport network. It did not compare this strategy to other interventions for expanding VL access, such as the placement of point-of-care devices in hard-to-reach facilities. Finally, we did not take into account patients’ own benefits and costs of dried sample use, such as fewer but faster switches to second-line treatment and potentially fewer clinic visits, nor possible efficiency gains available to other programs through the use of the other available spots on the PSC for testing.

In conclusion, adopting the partial use of dried specimens for further scale-up of VL monitoring programs for low volume and more difficult-to-reach sites will help achieve improved VL access for patients at the lowest cost per correct result. A 46% reduction in the price of the PSC would also make its use less costly in the short term in the partial PSC scenario compared to the partial DBS scenario. Further head-to-head field evaluations on the relative misclassification of PSC and DBS are required to further validate these conclusions.

## Supplementary Material

ciz338_Suppl_Supplementary_Appendix

## Notes

***Acknowledgments.*** The authors thank all stakeholders from the Zambian Ministry of Health, and members of the Zambian Laboratory Technical Working Group for contributions to the model development and for the refining of model assumption. The authors also thank Dr Lara Vojnov for her contributions to the updated dried blood spot misclassification rates.

***Financial support.*** This work was supported by USAID (cooperative agreement AID-OAA-A-15-00070) and by the Bill & Melinda Gates Foundation through the Innovation in Laboratory Engineered Accelerated Diagnostics investment (grant number OPP1171455). This work was also facilitated by the Providence/Boston Center for AIDS Research (grant number P3AI042853).

***Potential conflicts of interest.*** B. E. N., S. J. G., L. N., L. S.-I., L. S., and W. S. have received grants from the Bill & Melinda Gates Foundation. B. E. N. and S. J. G. have received grants from USAID. S. C. has received funding from Roche Diagnostics International AG (Rotkreuz, Switzerland) for partial funding of the analytical performance of the plasma separation card (see doi:10.1128/JCM.01336-18), and is a full-time employee of the National Health Laboratory Service, Johannesburg, South Africa. All other authors report no potential conflicts of interest. All authors have submitted the ICMJE Form for Disclosure of Potential Conflicts of Interest. Conflicts that the editors consider relevant to the content of the manuscript have been disclosed.
