# Supplementary material for: Cost and Impact of Dried Blood Spot Versus Plasma Separation Card for Scale-up of Viral Load Testing in Resource-limited Settings
Source: Clin Infect Dis. 2019 Apr 26;70(6):1014–20. doi: 10.1093/cid/ciz338 (PMC7931834; doi:10.1093/cid/ciz338)
Supplement: ciz338_Suppl_Supplementary_Appendix [file ciz338_suppl_supplementary_appendix.docx]

**Supplementary Appendix**

**Text S1. Costing**

**A.** *Viral load costing*

The cost per viral load test for a centralized test setting was estimated using the Testing Platform Cost Model (TPCM) developed by HE^2^RO staff ([www.heroza.org/tools](http://www.heroza.org/tools)). This cost model provides a simple tool for analysts and decision-makers to determine the cost per test for a testing platform from a provider’s perspective. The cost to a provider of a test depends on many individual components. These include the material costs per test, the salaries of staff who conduct start-up and daily quality control activities on the machine, the salaries of staff who perform the test, how the technology is integrated into patient flow and management, equipment and other related costs (e.g. insurance, maintenance and training), the expected working life of the platform itself, and the opportunity cost of funds used to acquire the platform (i.e. the discount rate).

This tool is used to calculate the cost per test using a hybrid of a bottom-up and ingredients-based costing approach for a Roche Cobas®Ampliprep/Cobas®TaqMan 96 (Roche Molecular Diagnostics, Branchburg, USA) platform (CAP/CTM 96). The cost per test using a plasma sample (using whole blood sample collection), a dried blood specimen (DBS) and a dried plasma separation (PSC) card is calculated.

Where prices were quoted in US dollars for previous years, they were inflated to the cost year (2018) using the consumer price index obtained from [www.usinflationcalculator.com](http://www.usinflationcalculator.com). For prices quoted in Zambian kwacha, the inflation rate was based on data from the Zambian Central statistical Office (Zambia Data Portal) and the exchange rate between the Zambian kwacha and the United States dollar was based on data from the Bank of Zambia. All costs are reported in US dollars and for 2018.

**Table S1** summarizes all the cost components by specimen type and the respective source tables.

**Table S1: Summary table: Viral load test cost using the Roche platform (US$)**

| ***Sample type*** | **plasma** | **DBS** | **PSC** |
| --- | --- | --- | --- |
| Sample collection | 1.18 (Table S2) | 1.39 (Table S3) | 5.47 (Table S3) |
| Sample analysis at lab | 17.22 (Table S4) | 17.54 (Table S4 +S5) | 17.54 (Table S4 +S5) |
| Total cost per viral load test | **18.40** | **18.93** | **23.01** |

**Table S2: Sample collection plasma: Cost per test– assumptions and sources**

| **Cost category** | **Unit cost (US$)** | **Items included** | **Assumptions** | **Sources** |
| --- | --- | --- | --- | --- |
| Materials | 0.31 | Spirits, cotton wool, syringe, needles, EDTA tubes, specimen bags, gloves, specimen containers, apron, waste bin liners, sharps container, chlorine | 5ml of spirits per patient, 0.5g cotton wool per patient, 2 waste bin liners are used per week, 1 apron per nurse per day, 1 disposable sharp box a day, 1 bottle of bleach a week for cleaning. These costs were allocated according to estimated numbers of patients seen for bloods a week. | Zambian Medical Stores Limited Catalogue 2016, and where prices not listed, equivalent material cost obtained through invoices and online quotes. |
| Staff | 0.87 | Nursing staff activities included drawing and collecting blood in an EDTA container. A cleaner’s time for cleaning surfaces and the room was also included. | Activity time estimations based on discussions with staff at CIDRZ lab (12.5 minutes per blood draw). | Government of Zambia salary scales.  CIDRZ laboratory, Lusaka, Zambia. |
| Overhead and Equipment costs | 0.001 | Overheads include electricity, water, Impressed government grant. Equipment includes desk, chair, specimen cooler box, dust bins, ice packs, lab log book, bathroom basin with faucet, tourniquet kit | Overheads allocated according to size of the blood draw room and then allocated according to the number of ART patient visits per month. Equipment allocated according to the number of ART patient visits per month. | CIDRZ laboratory, Lusaka, Zambia. Invoices and quotes. |
| **Total** | **1.18** |  |  |  |

**Table S3: Sample collection for dried specimens: Cost per test– assumptions and sources**

| **Cost category** | **Unit cost (US$)** | **Items included** | **Assumptions** | **Sources** |
| --- | --- | --- | --- | --- |
| Roche DBS collection kit | 0.92 | Roche DBS blood collection kit | Assumed this contained alcohol swab, lancet, gauze/cotton wool, 2* DBS cards, 3*Sillica gel Sachets, 2*gloves, 1*humidity indicator, capillary tube | Zambian Medical Stores Limited Catalogue 2016 |
| Roche PSC collection kit | 5.00 | Roche PSC collection kit | Assumed worst case scenario of $5. Kit inclusive of lancet, capillary tube, sterilizer, card, and transport bag with desiccant. | The cost of the Roche PSC kit is based on personal email communication with Roche (24 January 2019). |
| Common materials | 0.09 | Hand sanitizer, bleach to wipe surfaces, band-aid, drying racks, apron, waste bin liners, sharps container. | Assumed a drying rack lasts a year, 2 waste bin liners are used per week, 1 apron per nurse per day, 1 disposable sharp box a day, 1 bottle of bleach a week for cleaning. These costs were allocated according to estimated numbers of patients seen for bloods a week. | Zambian Medical Stores Limited Catalogue 2016, and where prices not listed, equivalent material cost obtained through invoices and online quotes. |
| Staff | 0.38 | Nursing staff activities included: pricking the finger for blood, and prepping the DBS/PSC card. A cleaner’s time for cleaning surfaces and the room was also included. | Activity time estimations based on discussions with study personnel at Kanyama clinic in Lusaka (using the Alere q for EID). Added in an additional 2 minutes of staff time for greeting patients and explaining test. Estimated cleaning time is 1 minute per test. | Government of Zambia salary scales.  Kanyama clinic, Lusaka |
| Overhead and Equipment costs | 0.001 | Overheads include electricity, water, Impressed government grant. Equipment includes desk, chair, specimen cooler box, dust bins, ice packs, lab log book, bathroom basin with faucet, tourniquet kit | Overheads allocated according to size of the blood draw room and then allocated according to the number of ART patient visits per month. Equipment allocated according to the number of ART patient visits per month. | CIDRZ laboratory, Lusaka, Zambia. Invoices and quotes. |
| **Total DBS** | **1.39** | | | |
| **Total PSC** | **5.47** | | | |

For this paper, we based our cost estimates for a viral load test conducted at a centralized laboratory on a bottom-up costing analysis conducted at the Centre for Infectious Disease Research in Zambia (CIDRZ) laboratory located in the Kalingalinga area of Lusaka, Zambia. It is the primary laboratory for most of Lusaka province and it serves as a reference laboratory for molecular diagnosis (HIV, DNA, PCR and viral load testing) for a number of neighboring provinces.  It processes and conducts testing on approximately 1600 blood samples per day (of which 400 are viral loads). Viral load testing is conducted using the two Roche Cobas®Ampliprep/ Cobas®TaqMan 96 (CAP/CTM 96) (Roche Molecular Diagnostics, Branchburg, USA). There are a number of shared activities across these blood sample tests (including dried blood spot) as all blood samples are sorted, registered and stored together – as such all shared costs from these activities was allocated across all blood samples. Shared costs are allocated according to the proportion of laboratory tests that the test under evaluation accounts for. Overhead costs are allocated according to the space allocated to the specific test under evaluation relative to the entire facility. All assumptions and sources are detailed in **Table S4** below.

**Table S4: Centralized viral load cost per test – assumptions and sources**

| **Cost category** | **Unit cost (US$)** | **Items included** | **Assumptions** | **Sources** |
| --- | --- | --- | --- | --- |
| Materials | 14.74 | Pipette tips, Reagents (including SPU for Cobas, Tip-k for Cobas ampliprep, S-tube input for Cobas, KIT CAP-G/CTM wash reagent) | Included the central Medical Stores Limited distribution margin of 1.5%. | Biogroup Zambia Limited and Medical Stores Limited Catalogue 2016 |
| Staff | 0.47 | Laboratory staff activities for viral load testing included: registering the sample/data entry; sample preparation and running; interpretation of the result/result delivery. Other shared activities conducted by laboratory assistants on all blood samples included sample sorting, centrifuging, aliquoting, archiving, searching, temperature monitoring etc. | Activity time estimations based on discussions with staff at the CIDRZ laboratory as well as laboratory records. Shared staff time allocated across all blood sample tests. | Government of Zambia salary scales.  CIDRZ laboratory, Lusaka |
| Quality control | 0.04 | Quality control activities conducted by a laboratory scientist every shift included start-up/equipment maintenance (including checking the buffer and control tubes, emptying waste). A cleaner also cleaned the room containing the viral load equipment every day. | Activity time estimations based on discussions with staff at the CIDRZ laboratory | Used Zambian government salaries for staff time; Medical Stores Limited Catalogue 2016; CIDRZ laboratory, Lusaka |
| Equipment | 0.88 | CAP/CTM 96 ($160,000), air-conditioners, refrigerators, fire extinguishers, computers, biosafety cabinets, chairs, freezers, tables generator, thermomixer, printers, uninterrupted power supply etc. | Estimated the working life of a CAP/CTM 96 (5 years); Estimated other equipment working life based on the South African Revenue Service write-off periods as well as discussions with laboratory personnel. All equipment annualized at 5% discount rate. Shared equipment for the laboratory allocated across all blood sample tests. | USAID procurement invoices, other invoices, CIDRZ laboratory |
| Other | 0.74 | Includes annual and upfront training for laboratory personnel; maintenance and insurance costs for equipment (e.g. CAP/CTM 96 and fridges/freezers), waste removal, dinner and transport allowance for extra shift work. | Viral loads contribute 40% of all lab waste (based on CIDRZ staff estimate) | Used Zambian government salaries for staff time for training, used invoices from CIDRZ lab for maintenance, insurance, staff per diems, and waste removal |
| Overhead | 0.35 | Electricity, security services, motor vehicles, overhead staff (e.g. other laboratory scientists, managers and maintenance staff) | Estimated electricity based on EQUIP office electricity bill. Allocated 20.4% of the total building size to viral load testing activities (based on space used to conduct the test as well as a proportion of shared space). | Discussions with the CIDRZ laboratory senior staff. |
| **Total** | **17.22** |  |  |  |

In order to use DBS or PSC on the Roche analyzer, we have used the same process for extracting plasma that is used for DBS samples (namely for EID). One full DBS circle from each specimen is placed in a 1.8 mL specimen tube, 1000 μL of specimen pre-extraction reagent is added followed by spinning in a thermomixer (Eppendorf AG, Hamburg, Germany) for 10 minutes at 56◦C (1). This additional step for DBS and PSC is incorporated into the shared staff activities and the pre-extraction reagent is added to the material cost. **Table S5** details the assumptions and sources that were used to calculate this cost.

**Table S5: Additional costs for dried specimens: cost per test – assumptions and sources**

| **Cost category** | **Unit cost (US$)** | **Items included** | **Assumptions** | **Sources** |
| --- | --- | --- | --- | --- |
| Additional sample extraction for dried plasma/blood specimens | 0.32 | Kit Cap-G/CTM Specimen Pre-extract Ruo Gpr | This is used for EID DBS, have assumed it would be similar for dried whole blood or plasma for viral load | Biogroup Zambia Limited |

**B**. *Transport costing*

Transport costs are based on a previously described analysis and included vehicle running costs incurred per kilometer of travel (fuel, maintenance and insurance), recurrent vehicle capital costs that need to be budgeted for annually, as well as personnel costs for operating the system (Ministry of Health salaries for drivers) (2). In the model, all high-volume facilities have viral load plasma samples collected daily but dried specimen samples are collected only twice weekly. Low volume facilities were considered for weekly sample transport when using plasma specimens; with the use of dried specimen use, sample transportation at low volume facilities was reduced to every two weeks (3). Costs of an expanded sample transport network to reach the additional 5% of patients (241 facilities) are included for the dried specimen scenarios(4).

**Text S2. Sensitivity analysis**

**A**. *Sensitivity analysis methods*

We conducted a multiple univariate sensitivity analysis of the key inputs: 1) a change in the price of a PSC from the current quoted price of $5 to cost of a DBS collection kit cost of approximately $1; 2) the worst and best case cost scenario for conducting a viral load test on a Roche platform for a dried specimen ($15.31 to $20.39); 3) patient access rates for low and high volume facilities (varied from 70% to 90%); 4) increasing the assumed patient access rate at low-volume facilities from 40% up to 70% in the plasma-only scenario, in the event that the patient access rate differential between low and high volume facilities is absolute at scale and the magnitude of difference does not remain; 5) varying the sensitivity and specificity of different specimen types, and utilizing the range of the sensitivity and specificity of PSC on the equipment that is likely to completely replace the current CAP/CTM technology, the Cobas 8800 (sensitivity 97% [95% CI 93-99%], specificity 97% [95% CI 95-99%])(5); 6) varying the true proportion of underlying virological load failure from 10% to 40%; and 7) a deviation from the guidelines whereby only a proportion (65%) of repeat viral loads are requested in the event of an unsuppressed viral load. The results from this and the bivariate sensitivity analysis are report in **Figure S1**.

**B**. *Sensitivity analysis results*

Univariate and bivariate sensitivity analyses (**Figure S1**) highlight the key cost input parameters of our model for the two mixed scenarios, plasma+DBS and plasma+PSC. Both the plasma+DBS and plasma+PSC scenarios are most sensitive to the true proportion of underlying viralogical failure, the performance of their specimen type (in particular, specificity for the plasma+DBS and sensitivity for plasma+PSC), and the Roche viral load test cost. The plasma+DBS scenario is very sensitive to a decrease in the specificity of DBS – a deterioration in the specificity of DBS from the expected value of 94% to 89% would result in the plasma+PSC being cost neutral relative to the plasma+DBS scenario ($31.62).

Increasing the assumed patient access rate at low-volume facilities from 40% up to 70% in the plasma-only scenario decreased the cost per accurate test from $29.92 to $29.55. Reducing the proportion of repeat viral loads requested in the event of an unsuppressed viral load from 100% to 65% decreased the cost per correct result in all scenarios by between 3.2%-4.4%. Similarly, increasing the true proportion of underlying virological failure from 14% to 40% increased the cost per correct result in all scenarios by between 18.9% to 19.6%.

Excluding all parameters that do not affect the scenarios differentially (the sample access rate at high volume facilities, the cost of a conducting a viral load test on a dried specimen, and the true underlying proportion of virological failure), the bivariate analysis, whilst expanding the upward risk around the base case compared to the univariate analysis, did not deviate substantially from the results obtained via univariate analysis. This is likely due to low correlation between the input variables. A deterioration in both the specificity and sensitivity of DBS for the plasma+DBS scenario results in a cost per correct result of $35.45, a 15% increase.

**Figure S1. Tornado diagram of univariate and bivariate sensitivity analysis: key cost drivers by partial adoption scenario**


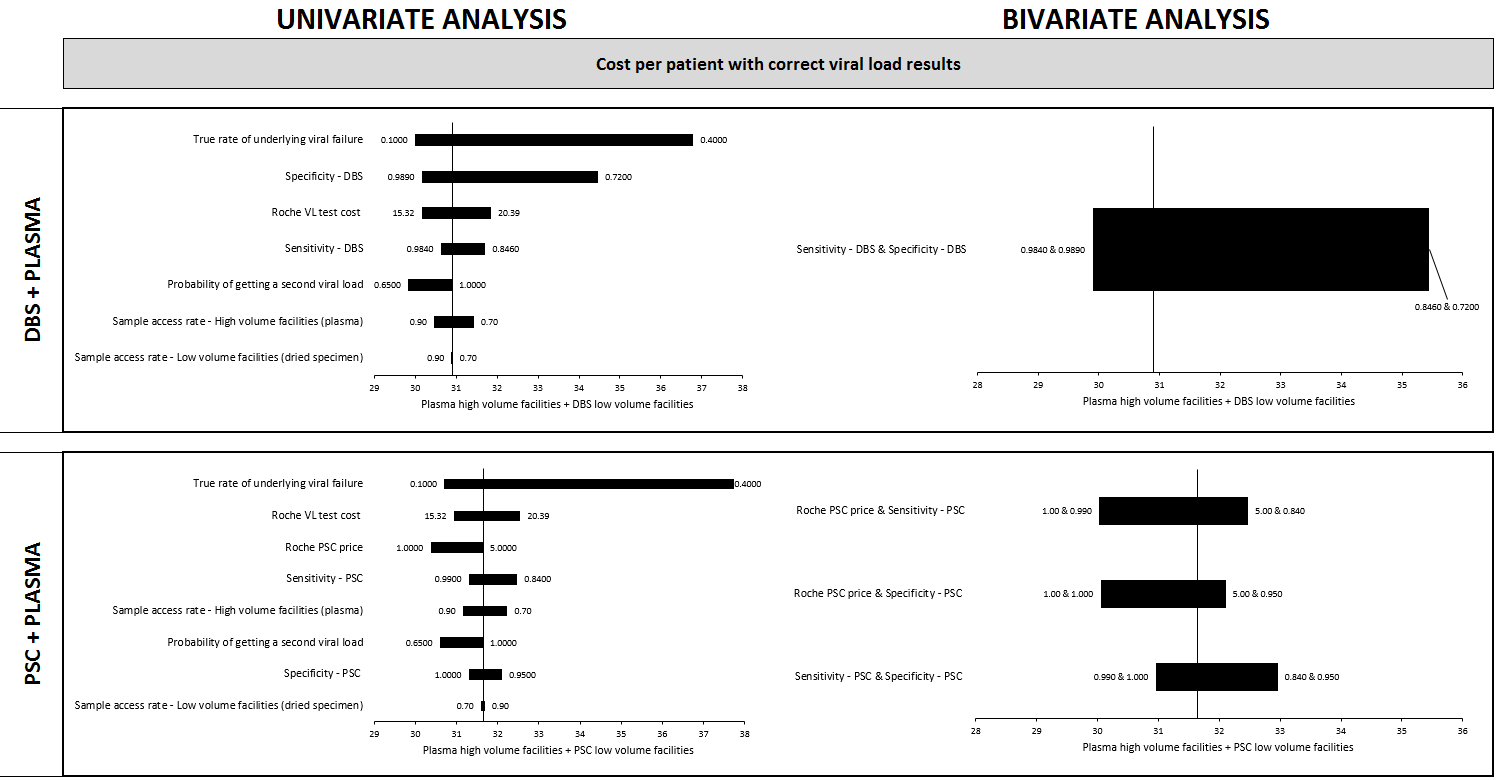


**Text S3. Head to head analysis**

We used estimates from the analysis of PSC in South Africa where the same samples were tested using both DBS and PSC with DBS being performed on the Abbott rt2000 platform and PSC being performed on the Cobas 6800/8800 and CAP/CTM (6). Whilst this is not a perfect head-to-head analysis (DBS is not conducted on the Cobas 6800/8800 or CAP/CTM), by using the same population with the same underlying distribution of viral load failure, it is a better comparison. Results from this analysis significantly strengthen the case for partial adoption of PSC with the plasma+PSC scenario outperforming the plasma+DBS scenario. **Table S6** presents the results for DBS on the Abbott rt2000 and PSC on the CAP/CTM. **Table S7** presents the results for DBS on the Abbott rt2000 and PSC on the Cobas 6800/8800.

**Table S6: Head to head analysis using DBS on Abbott and PSC on CAP/CTM**

|  | ***Potential access with only plasma specimen use*** | | ***Potential access with dried specimen use*** | | | | | | | |
| --- | --- | --- | --- | --- | --- | --- | --- | --- | --- | --- |
|  | Plasma only | | Plasma high volume + DBS low volume | | Plasma high volume + PSC low volume | | DBS only for whole VL system | | PSC only for whole VL system | |
|  | n | cost | n | cost | n | cost | n | cost | n | cost |
| Number of patients accessing viral load tests per year (% total) | 814 066 (65%) | | 965 587 (77%) | | 965 587 (77%) | | 965 587 (77%) | | 965 587 (77%) | |
| Number of ART facilities reached (% total) | 800 (54%) | | 1 041 (71%) | | 1 041 (71%) | | 1 041 (71%) | | 1 041 (71%) | |
| Number of patients with correct viral load results | 795 342 |  | 881 426 |  | 923 585 |  | 710 672 |  | 869 028 |  |
| Viral loads on dried specimens | - | $0 | 323 404 | $6 122 044 | 292 382 | $6 727 712 | 1 214 785 | $22 995 889 | 1 098 258 | $25 270 928 |
| Viral loads on plasma specimens | 930 982 | $17 130 067 | 810 283 | $14 909 215 | 810 283 | $14 909 215 | - | $0 | - | $0 |
| Number of facility visits required | 931 011 | $3 398 191 | 1 133 688 | $4 155 408 | 1 102 666 | $4 024 916 | 1 214 785 | $4 499 154 | 1 098 258 | $4 008 996 |
| Transport cost |  | $3 264 509 |  | $3 637 168 |  | $3 637 168 |  | $2 390 317 |  | $2 390 317 |
| Annual system cost |  | $23 792 767 |  | $28 823 834 |  | $29 299 011 |  | $29 885 361 |  | $31 670 241 |
| ***Cost per patient with a viral load result*** |  | *$29.23* |  | *$29.85* |  | *$30.34* |  | *$30.95* |  | *$32.80* |
| ***Annual cost per patient with correct viral load results*** |  | ***$29.92*** |  | ***$32.70*** |  | ***$31.72*** |  | ***$42.05*** |  | ***$36.44*** |
| ***Average annual cost per additional patient with correct viral load results compared to plasma-only*** |  |  |  | *Weakly dominated* |  | *$42.94* |  | *Dominated* |  | *Dominated* |

**Table S7: Head to head analysis using DBS on Abbott and PSC on Cobas 6800/8800**

|  | ***Potential access with only plasma specimen use*** | | ***Potential access with dried specimen use*** | | | | | | | |
| --- | --- | --- | --- | --- | --- | --- | --- | --- | --- | --- |
|  | Plasma only | | Plasma high volume + DBS low volume | | Plasma high volume + PSC low volume | | DBS only for whole VL system | | PSC only for whole VL system | |
|  | n | cost | n | cost | n | cost | n | cost | n | cost |
| Number of patients accessing viral load tests per year (% total) | 814 066 (65%) | | 965 587 (77%) | | 965 587 (77%) | | 965 587 (77%) | | 965 587 (77%) | |
| Number of ART facilities reached (% total) | 800 (54%) | | 1 041 (71%) | | 1 041 (71%) | | 1 041 (71%) | | 1 041 (71%) | |
| Number of patients with correct viral load results | 795 342 |  | 881 426 |  | 934 381 |  | 710 672 |  | 909 583 |  |
| Viral loads on dried specimens | - | $0 | 323 404 | $6 122 044 | 299 169 | $6 883 868 | 1 214 785 | $22 995 889 | 1 123 750 | $25 857 487 |
| Viral loads on plasma specimens | 930 982 | $17 130 067 | 810 283 | $14 909 215 | 810 283 | $14 909 215 | - | $0 | - | $0 |
| Number of facility visits required | 931 011 | $3 398 191 | 1 133 688 | $4 155 408 | 1 109 452 | $4 050 328 | 1 214 785 | $4 499 154 | 1 123 750 | $4 104 451 |
| Transport cost |  | $3 264 509 |  | $3 637 168 |  | $3 637 168 |  | $2 390 317 |  | $2 390 317 |
| Annual system cost |  | $23 792 767 |  | $28 823 834 |  | $29 480 579 |  | $29 885 361 |  | $32 352 255 |
| ***Cost per patient with a viral load result*** |  | *$29.23* |  | *$29.85* |  | *$30.53* |  | *$30.95* |  | *$33.51* |
| ***Annual cost per patient with correct viral load results*** |  | ***$29.92*** |  | ***$32.70*** |  | ***$31.55*** |  | ***$42.05*** |  | ***$35.57*** |
| ***Average annual cost per additional patient with correct viral load results compared to plasma-only*** |  |  |  | *Weakly dominated* |  | *$40.91* |  | *Dominated* |  | *Dominated* |

**References**

1. Zeh C, Ndiege K, Inzaule S, Achieng R, Williamson J, Chang JCW, et al. Evaluation of the performance of Abbott m2000 and Roche COBAS Ampliprep/COBAS Taqman assays for HIV-1 viral load determination using dried blood spots and dried plasma spots in Kenya. PLoS One. 2017;12(6):1–15.

2. Nichols B, Girdwood S, Crompton T, Stewart-Isherwood L, Berrie L, Chimhamhiwa D, et al. Sustainable viral load monitoring scale-up: geospatial optimisation model for Zambia. In: Conference on Retroviruses and Opportunistic Infections 2018. 2018.

3. Aitken SC, Wallis CL, Stevens W, de Wit TR, Schuurman R. Stability of HIV-1 Nucleic Acids in Dried Blood Spot Samples for HIV-1 Drug Resistance Genotyping. Paxton WA, editor. PLoS One [Internet]. Public Library of Science; 2015 Jul 6 [cited 2018 Nov 13];10(7):e0131541. Available from: http://dx.plos.org/10.1371/journal.pone.0131541

4. Nichols B, Girdwood S, Crompton T, Stewart-Isherwood L, Berrie L, Chimhamhiwa D, et al. Monitoring viral load for the last mile: What will it cost? In: International AIDS conference. 2018.

5. Carmona S, Seiverth B, Magubane D, Hans L, Hoppler M. Separation of plasma from whole blood using the cobas Plasma Separation Card: a compelling alternative to dried blood spots for the quantification of HIV-1 viral load. J Clin Microbiol. 2019;(February).

6. Carmona S, Seiverth B, Magubane D, Hans L, Hoppler M. A compelling alternative to dried blood spots- plasma separation card for the quantification of HIV-1RNA viral load. In: AIDS, Amsterdam, International AIDS Society. 2018.
